# Rate and predictors of 30-day readmission for *clostridiodes difficile*: a United States analysis

**DOI:** 10.1080/07853890.2021.2023211

**Published:** 2022-01-06

**Authors:** Asim Kichloo, Zain El-amir, Dushyant Singh Dahiya, Mohammad Al-Haddad, Jagmeet Singh, Gurdeep Singh, Carlos Corpuz, Hafeez Shaka

**Affiliations:** a Department of Internal Medicine, Central Michigan University College of Medicine, Saginaw, MI, USA; b Department of Internal Medicine, Samaritan Medical Center, Watertown, NY, USA; c Division of Gastroenterology and Hepatology, Department of Internal Medicine, Indiana University School of Medicine, Indianapolis, IN, USA; d Department of Nephrology, Guthrie Robert Packer Hospital, Sayre, PA, USA; e Department of Medicine and Endocrinology, Our Lady of Lourdes Memorial Hospital, Binghamton, NY, USA; f Department of Internal Medicine, John H. Stronger Jr. Hospital of Cook County, Chicago, IL, USA

**Keywords:** *Clostridiodes difficile* Enterocolitis, readmission, epidemiology predictors, mortality costs

## Abstract

**Background:**

*Clostridiodes difficile* is a leading cause of healthcare-associated diarrhea. In this study, we aimed to identify the rates and predictors for 30-day readmissions of *Clostridiodes difficile* Enterocolitis (CDE) in the United States.

**Methods:**

We conducted a retrospective study of the Nationwide Readmissions Database to identify adult hospitalizations with a principal diagnosis of CDE for 2018. Individuals <18 years old and elective hospitalizations were excluded. Primary outcomes included readmission rate and the top ten principal diagnosis on readmission, while the secondary outcomes were inpatient mortality, hospital costs and independent predictors of 30-day all-cause readmissions. Furthermore, we devised a scoring system to estimate the risk of CDE readmissions. Stata® Version 16 was used for statistical analysis and *p*-values ≤0.05 were statistically significant.

**Results:**

We identified 94,668 index hospitalizations and 18,296 readmissions at 30-days for CDE in 2018. The 30-day all-cause readmission rate was 25.7%. On readmission, CDE was the most common principal diagnosis (25.7%), followed by unspecified sepsis, and acute renal failure. A female predominance was also noted for index and 30-day readmissions of CDE. Compared to index admissions, we noted higher odds of inpatient mortality [4.4 vs 1.4%, Odds Ratio (OR):3.32, 95% Confidence Interval (CI):2.87–3.84, *p* < 0.001], longer mean length of stay (LOS) [6.4 vs 5.6 days, Mean Difference (MD):0.9, 95% CI:0.7–1.0, *p* < 0.001), and higher mean total hospital charge (THC) [$56,015 vs $40,871, MD:15,144, 95% CI:13,260–17,027, *p* < 0.001] for 30-day readmissions of CDE. Independent predictors for 30-day all-cause readmissions of CDE included discharged against medical advice (AMA) [Adjusd Hazard Ratio (aHR):2.01, 95% CI:1.73–2.53, *p* < 0.001], diabetes mellitus (DM) [aHR:1.22, 95% CI:1.16–1.29, *p* < 0.001], and chronic kidney disease (CKD) [aHR:1.29, 95% CI:1.21–1.37, *p* < 0.001].

**Conclusion:**

The all-cause 30-day readmission rate and inpatient mortality for CDE was 25.7% and 4.4%, respectively. Discharge AMA, DM and CKD were independent predictors for 30-day all-cause readmissions of CDE.

**KEY MESSAGE**
1. The 30-day all-cause readmission rate for *Clostridiodes difficile* Enterocolitis was noted to be 21.4% in 2018.
2. Independent predictors of 30-day all-cause readmissions for *Clostridiodes difficile* Enterocolitis include diabetes mellitus, discharged against medical advice and chronic kidney disease.
3. Readmissions of *Clostridiodes difficile* Enterocolitis had higher mortality rates, healthcare cost and length of hospital stay compared to index admissions.

## Introduction

*Clostridiodes difficile* is a gram-positive, spore-forming, anaerobic bacillus that can lead to diarrhea and/or enterocolitis of varying severity [1]. It is closely associated with antibiotic use and has been identified as the leading cause of healthcare-associated diarrhea in the United States (US) with literature reporting a 200% increase in *C. difficile* related hospitalizations between 2000 and 2009 [2–4]. Furthermore, studies continue to report rising rates of incidence, severity, and mortality associated with *C. difficile* [4]. From a hospitalization perspective, it has been estimated that over 200,000 individuals require hospitalization for *C. difficle*-associated diarrhea every year accounting for 1% of all US hospitalizations [5,6]. Hence, it is well established that CDE places a significant burden on the US healthcare system in terms of costs (over $4 billion at acute care facilities) and resource utilization [2,7]. Additionally, CDE is also a major health risk to individual patients, particularly those admitted to the hospital, and may affect the patients’ overall quality of life [8].

Readmission of CDE have been thoroughly investigated; however, there continue to be significant gaps in current literature. Prior studies have focussed their attention on the characteristics and associations of readmissions, but do not provide additional information on key hospitalization characteristics and predictors of readmissions. Identification of these variables may help reduce readmissions rates, inpatient mortality, and the burden of CDE on the US healthcare system [5,9]. Hence, we designed this retrospective study to determine hospitalization characteristics and adverse outcomes of 30-day readmissions of CDE, and compare them to index admissions to identify key differences. Furthermore, we also developed a scoring system to estimate the risk of CDE readmissions.

## Materials and methods

### Design and data source

We conducted a retrospective study from the Nationwide Readmissions Database (NRD) to identify adult hospitalizations with a principal diagnosis of CDE. The NRD is the largest, publicly available, multi-ethnic, all-payer inpatient healthcare readmission database in the US, drawn from the Agency for Healthcare Research and Quality (AHRQ) Healthcare Cost and Utilization Project (HCUP) State Inpatient Databases (SID). It is an annual file constructed using one calendar year of discharge data. The discharges included in the study were treated at hospitals across the US (excluding rehabilitation or long-term acute care hospitals) and had patient linkage numbers that were verified and used to identify the study population. Discharge weights were calculated using post-stratification for hospital characteristics (census region, urban/rural location, teaching status, bed size, and hospital control) and patient characteristics (sex and five age groups [0, 1–17, 18–44, 45–64, and 65 and older]) [10]. For the 2018 calendar year, the NRD had discharge data from 28 geographically dispersed states accounting for 59.7% of the total US resident population and 58.7% of all US hospitalizations [10]. The database is validated by HCUP and designed to support readmission analyses on a national scale as the weighted analysis allows us to obtain 100% of the US hospitalizations within a given year [10]. In the NRD, up to 40 discharge diagnoses and 25 procedures are collected for each patient using the International Classification of Diseases, Tenth Revision, Clinical Modification (ICD10-CM/PCS) for each calendar year. The diagnoses are classified as the principal diagnosis, which is the primary reason for hospitalization, and secondary diagnosis, which includes any other discharge diagnosis.

### Study population

The study involved all adult (≥18 years) hospitalizations with a primary diagnosis of CDE using the ICD-10 diagnostic codes (A04.7X). The CDE types in this diagnostic code included patients with recurrent CDE (A04.71) and CDE without mention of recurrence (A04.72). Individuals <18 years of age and elective hospitalizations were excluded. Furthermore, we excluded hospitalizations in December due to the lack of an adjoining 30-day period to assess 30-day readmissions. Using the unique hospitalization identifiers available within the NRD, we identified index hospitalizations, and one subsequent hospitalization within 30 days tagged as readmission for CDE.

### Outcome measures

The primary outcomes included the rates of 30-day readmissions and the top ten principal diagnosis on readmission for 30-day readmission of CDE. Moreover, the secondary outcomes were inpatient mortality, mean length of stay (LOS), hospital costs [mean total healthcare cost (THC) and mean cost of hospitalization (COH)], and independent predictors for 30-day all-cause readmissions of CDE. Furthermore, we also developed a readmission risk scoring system based on these independent predictors to estimate the risk of CDE readmissions.

### Statistical analysis

The data was analysed using Stata^®^ Version 16 software (StataCorp, Texas, USA). We conducted all the analysis using the weighted samples for national estimates in adjunct with HCUP regulations for using the NRD. Age grouped as 18–39 years represented young adults, 40–64 years represented middle-aged adults, and ≥65 years represented the elderly.

The comorbidity burden was assessed using the Sundararajan’s adaptation of the modified Deyo’s Charlson comorbidity index (CCI) [11]. The CCI contains a list of comorbidities with an assigned weighted score based on the relative risk of 1-year mortality. Consequently, the sum of the index score is an indicator of disease burden and an excellent estimator of mortality. The modified Deyo’s CCI is classified into four groups with increasing risk for mortality and has been adapted in population-based research [11]. A score of >3 has about a 25% 10-year mortality, while a score of 2 or 1 has a 10% and 4% 10-year mortality, respectively. This cut-off point was chosen as a means of assessment of the increased risk of mortality [11,12].

For the comparative analysis, we used the Pearson’s chi-square test to compare the characteristics between the index hospitalization and readmissions. A univariable logistic regression was employed to compare readmission mortality, and linear regression model was used to compare readmission, LOS, THC, and COH to the index hospitalization. Furthermore, we employed a univariable pre-screening analysis to identify variables associated with readmission and potential predictors of 30-day all-cause readmission. Additionally, we identified mean age, sex, hospital location, hospital teaching status, hospital bed size, mean household income, and the comorbidities for CDE readmissions. Variables with p-value less than 0.2 were included in the final multivariable regression analysis. Subsequently, we performed a multivariable Cox regression analysis to identify independent predictors of readmissions with *p*-values <0.05 set as the threshold for statistical significance. This model included CDE type (CDE with or without mention of recurrence), sex, discharg against medical advice (AMA), insurance status, hospital teaching status, hospital location, Hypertension, Chronic Obstructive Pulmonary Disease (COPD), Diabetes Mellitus (DM), Chronic Kidney Disease (CKD), Protein-Energy Malnutrition (PEM), and history of neoplasm.

**Figure 1.**
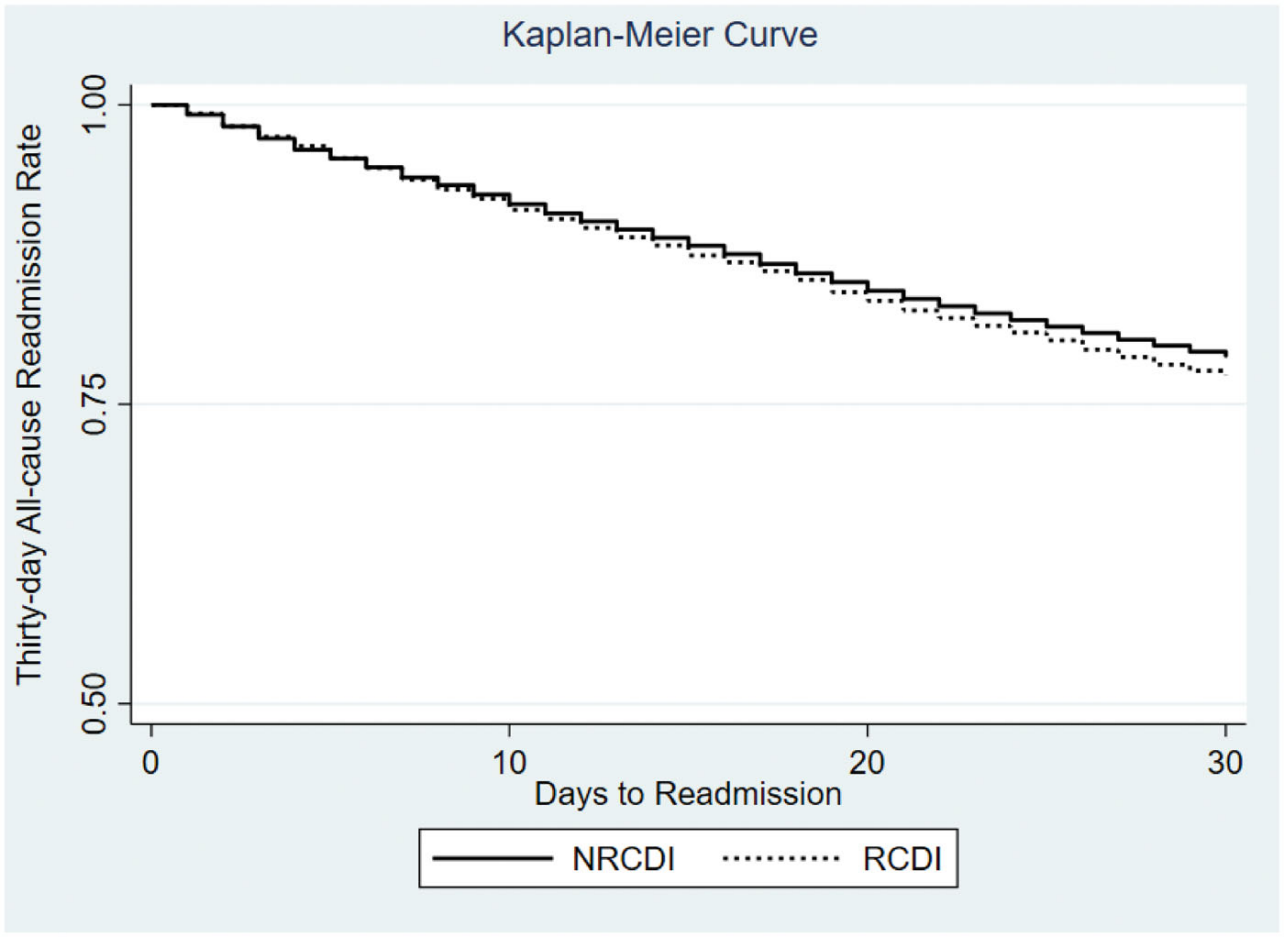
Kaplan-Meier survival estimates for 30-day all-cause readmission *Clostridiodes difficile* Enterocolitis (CDE) by type in the United States. NRCDI: Non-recurrent *Clostridiodes difficile* infection; RCDI: Recurrent *Clostridiodes difficile* infection.

Furthermore, we selected independent predictors which have >10% increased hazard ratio for 30-day readmission to develop a risk scoring system following index CDE hospitalizations. Variables such as DM, COPD, CKD, PEM, and AMA were included. A score of 1 was assigned to these five variables and the incidence rate for readmission for aggregate scores were obtained. Finally, a Kaplan-Meier curve (Figure 1) was generated based on the findings of our study.

### Ethical considerations

This was a database study using the NRD. As NRD lacks specific patient identifiers, our study was exempt from Institutional Review Board (IRB) approval as per guidelines put forth by our institutional IRB for analysis of HCUP databases.

## Results

### Rate and top ten principal diagnosis for 30-Day readmissions of CDE

For 2018, there were 94,668 index hospitalizations and 18,296 readmissions at 30-days for CDE. The 30-day all-cause readmission rate was noted to be 25.7%. On readmission, CDE was identified as the most common principal diagnosis (25.7%), followed by unspecified sepsis (11.1%), acute renal failure (2.9%), urinary tract infections (unspecified site) [1.7%], and dehydration (0.9%) (Tables 1 and 2).

**Table 1.** Top principal diagnoses based on International Classification of Diseases (ICD)-10 diagnostic chapters for 30-day readmissions of *Clostridiodes difficile* Enterocolitis in the United States.

Principal Readmission Diagnosis Based on ICD-10 Diagnostic Chapters	Proportion (%)
Certain Infectious and Parasitic Diseases	41.7
Diseases of the Digestive System	14.4
Diseases of the Circulatory system	11.2
Diseases of the Genitourinary System	6.5
Endocrine, Nutritional and Metabolic Diseases	5.7
Diseases of the Respiratory system	5.6
Neoplasms	2.1
Mental, Behavioural and Neurodevelopmental Disorders	2.0
Diseases of the Nervous System	1.8
Diseases of the Musculoskeletal System and Connective Tissue Diseases	1.4
Diseases of the Skin and Subcutaneous Tissue	1.3
Diseases of Blood and Blood-Forming Organs	1.1

ICD: International Classification of Diseases

**Table 2.** Top ten principal diagnoses for 30-day readmissions of *Clostridiodes difficile* Enterocolitis in the United States.

Principal readmission diagnosis	Proportion (%)
Clostridium Difficile Enterocolitis	25.7
Unspecified Sepsis	11.1
Acute Renal Failure	2.9
Hypertensive Heart and Chronic Kidney Disease with Heart Failure	2.3
Urinary Tract Infection (site not specified)	1.7
Pneumonia (unspecified organism)	1.4
Hypertensive Heart Disease with Heart Failure	1.3
Sepsis secondary to Anaerobes	1.1
Noninfective Gastroenteritis and Colitis (unspecified)	1.1
Dehydration	0.9

### Comparative analysis of hospitalization characteristics for index admission and 30-Day readmissions of CDE

Compared to index admissions, 30-day readmissions of CDE had a lower proportion of females (61.8 vs. 64.1%, *p* < 0.001) without a statistically significant difference in mean age. Thirty-day readmissions of CDE had a higher proportion of patients with CCI score ≥3 (50.5 vs. 38.1%, *p <* 0.001) compared to index admissions. Furthermore, there were a higher portion of patients with comorbidities such as DM, congestive heart failure (CHF), CKD and COPD in the 30-day readmission cohort compared to index admissions.

### Comparative analysis of clinical outcomes for index admissions and 30-Day readmissions of CDE

From a mortality perspective, 30-day readmissions of CDE were associated with significantly higher odds of inpatient mortality (4.4 vs 1.4%, OR:3.32, 95% CI:2.87–3.84, *p* < 0.001) compared to index admissions. Additionally, these readmissions also had longer mean LOS, and higher THC and COH (Table 3).

**Table 3.** Comparative analysis of clinical outcomes for index admissions and 30-day readmissions of *Clostridiodes difficile* enterocolitis in the United States.

Outcome	Index admission	30-day readmission	Odds ratio (95% ci)	*p*-value
In-patient Mortality (%)	1.4	4.4	3.32 (2.87–3.84)	<.001
Mean Length of Stay (days)	5.6	6.4	0.9^#^ (0.7–1.0)	<.001
Mean Total Hospital Charges (USD)	40,871	56,015	15,144^#^ (13260–17027)	<.001
Mean Total Hospital Cost (USD)	10,064	13,504	3,439^#^ (1486–2101)	<.001

^#^Mean difference, CI: confidence interval.

### Predictors for 30-day all-cause readmissions of CDE

**Figure 2.**
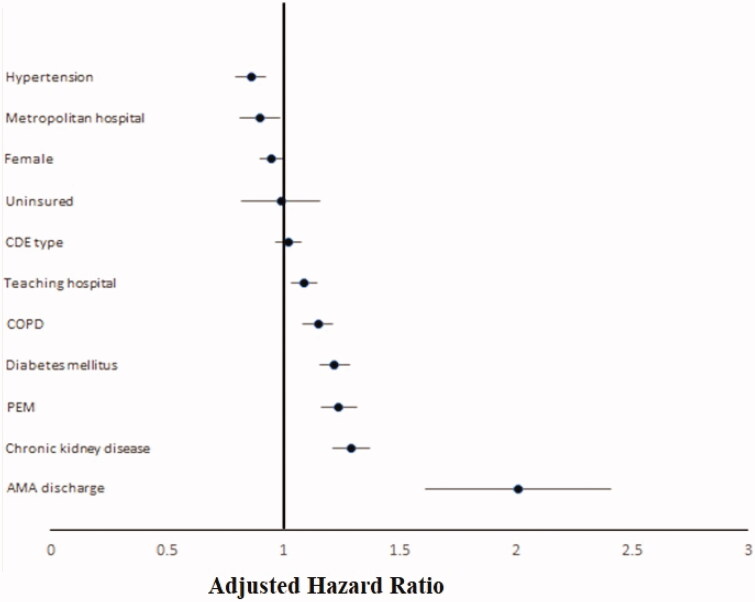
Forrest plot showing independent predictors of 30-day all-cause readmissions of *Clostridiodes difficile* Enterocolitis in the United States. AMA: Against Medical Advice; CDE: *Clostridiodes difficile* enterocolitis; COPD: Chronic Obstructive Chronic Disease; PEM: Protein-Energy Malnutrition.

Independent predictors for 30-day all-cause readmissions of CDE included discharge AMA (aHR:2.01, 95% CI:1.73–2.53, *p* < 0.001), DM (aHR:1.22, 95% CI:1.16–1.29, *p* < 0.001), and CKD (aHR:1.29, 95% CI:1.21–1.37, *p* < 0.001) [Table 4 and Figure 2].

**Table 4.** Hospitalization characteristics for index admissions and 30-day readmissions of *Clostridiodes difficile* Enterocolitis in the United States.

Variable	Index admission (%)	Thirty-day readmission (%)	*p*-value
Total Number of Hospitalizations	94,668	18,296	
Patient characteristics			
Age (mean years) ± SE	66.4 ± 0.3	66.4 ±.4	.907
Women (%)	64.1	61.8	<.001
Charlson Comorbidity Index Score (%)			<.001
0	25.3	15.6	
1	19.7	16.9	
2	16.9	17.0	
≥3	38.1	50.5	
Insurance Type (%)			<.001
Medicare	69.2	71.6	
Medicaid	10.3	11.9	
Private	18.2	14.4	
Uninsured	2.3	2.1	
Median Annual Income in Patient’s Zip Code* (USD)			.114
1–45,999	27.6	28.7	
46,000–58,999	29.7	29.6	
59,000–78,999	25.0	24.6	
≥79,000	17.7	17.1	
Comorbidities			
Hypertension	37.5	31.6	<.001
Diabetes Mellitus	30.0	33.3	<.001
Smoking History	14.3	13.5	.046
Congestive Heart Failure	18.1	22.7	<.001
Chronic Kidney Disease	27.1	31.4	<.001
Obesity	12.9	12.9	.961
Dyslipidemia	38.9	38.9	.952
Coronary Artery Disease	23.7	27.5	<.001
Prior Cerebrovascular Accident	2.8	3.4	.003
Protein-Energy Malnutrition	14.8	20.0	<.001
Chronic Obstructive Pulmonary Disease	17.6	21.3	<.001
History of Neoplasm	14.4	17.7	<.001
Hospital characteristics			
Hospital Bed Size			<.001
Small	19.3	17.7	
Medium	27.5	25.9	
Large	53.2	56.4	
Metropolitan Location	76.2	77.5	.010
Teaching Status	65.3	68.6	<.001

### Kichloo’s score for 30-Day readmission following CDE hospitalization

Five independent predictors of 30-day readmission of CDE, namely DM, COPD, CKD, PEM, and discharge AMA, were used to develop the *Kichloo’s* scoring system. CDE hospitalizations with a score of 0 had a 17.9% rate of readmission, while hospitalizations with a score of ≥3 had a readmission rate of 36.8% (Table 5). The score was structured into average risk (0), and high risk (≥1) for 30-day readmission.

**Table 5.** Risk assessment model for 30-day readmissions of *Clostridiodes difficile* Enterocolitis in the United States using the Kichloo Scoring System.

Kichloo Score	Incidence Rate Per Day Per 1,000 Hospitalizations	30-Day Readmission Incidence Rate (%)	Index Admission (%)	Readmission (%)
0	6	17.9	40.9	32.5
1	9	25.8	33.9	37.6
2	11	32.4	19.9	23.5
≥3	13	36.8	5.3	5.4

**Kichloo Score** [readmission risk following *Clostridiodes difficile* enterocolitis (CDE) hospitalizations]: One point each for Diabtes Mellitus, Chronic Obstructive Pulmonary Disease, Chronic Kidney Disease, Protein Energy Malnutrition, and Discharge Against Medical Advice.

## Discussion

### Readmission rate and top ten principal diagnosis for 30-Day readmissions of CDE

The 30-day all-cause readmission rate for CDE was noted to be 25.7%. This high readmission rate may be due to the fact that patients with a history of *C. difficile* infections tend to have a higher degree of relapse and therefore readmission. We also noted that at the time of readmission, CDE was the most common principal diagnosis. This finding aligns with current literature which reports that patients with recurrent *C. difficile* infections are more likely to be readmitted and have longer lengths of hospitalizations compared to patients without a recurrent CDE infection [9,13]. Furthermore, other top common principal diagnosis on readmission included unspecified sepsis, and acute renal failure. The relationship between sepsis and CDE is relatively well studied with literature reporting increased risk of severe sepsis in patients with CDE [14–16]. Moreover, acute renal failure, which was also noted in our study, may be a consequence of volume depletion secondary to watery diarrhea and may increase the odds of subsequent readmissions [17].

### Hospitalization characteristics of index admissions and 30-Day readmissions of CDE

We noted that 30-day readmissions of CDE had a higher proportion of patients with comorbidities such as DM, CHF, CKD, and COPD compared to the index admissions. The association between several of these comorbidities with CDE has already been established in literature. For example, patients with CDE may have prolonged periods of continued watery diarrhea leading to volume depletion and progression of pre-renal acute kidney injury to acute tubular necrosis and ultimately CKD [17]. Additionally, these patients with underlying CKD may be at a higher risk of developing acute on chronic kidney failure due to significant dehydration secondary to diarrhea. CDE has also been linked to acute renal failure due to the action of toxin B on the collecting duct leading to increased loss of volume and subsequent death [18]. Additionally, *C. difficile* infection has also been linked to the development of immunoglobulin A (IgA) nephropathy in patients with a prior diagnosis of acute and chronic renal failure [18]. Hence, through multiple mechanisms, CDE may lead to readmission in patients with pre-existing renal disease. Furthermore, per guidelines, for patients with inflammatory bowel disease, renal failure, DM, and haematologic malignancies, who present to the hospital for acute-onset diarrhea, screening studies for *C. difficile* are recommended as they are prominent risk factor for index or recurrent infection [19,20].

Thirty-day readmissions of CDE also had a higher proportion of patients with CCI score ≥3 compared to index admissions reflecting a higher comorbidity burden in these patients. Hence, we advocate for the need of increased surveillance for *C. difficile* in patients with multiple comorbidities as they may be at higher-than-average risk of acquiring CDE and subsequent readmissions after CDE.

### Comparison of clinical outcomes for index admission and 30-Day readmissions, and predictors of 30-day readmissions of CDE

Compared to index admissions, 30-day readmissions of CDE were associated with significantly higher odds of inpatient mortality (Table 3). These readmissions were also associated with increased mean LOS, and higher THC and COH (Table 3). These findings reflected current literature as CDE is associated with increased LOS, THC, and risk of readmissions [5]. Higher hospital costs and longer length of hospital stay may, in part, be due to the need of subspeciality care and a higher prevalence of comorbidities in these patients which may require an interdisciplinary team and additional resources for management [21]. Moreover, readmissions within 60 days of an initial *C. difficile* infection with a secondary diagnosis of recurrent *C. difficile* has also been shown to have poor clinical outcomes and high healthcare costs, highlighting the healthcare burden that the infection poses [22].

In this study, independent predictors for 30-day all-cause readmissions of CDE included discharge AMA, DM, PEM, COPD and CKD. In an attempt to identify individuals at the highest risk of readmissions and thereby inpatient mortality, we developed a scoring system (*Kichloo’s Score*) for CDE readmissions. We used these independent risk factors for our scoring system and one point each was given for DM, COPD, CKD, PEM, and AMA. We then calculated the risk of readmissions for these patients based on the assigned scores. We noted that patients with a score of 0 had a readmission rate of 17.9%, while patients with a score of ≥3 had a readmission rate of 36.8% (Table 5). We believe that this scoring system may help physicians identify and closely monitor individuals with the highest risk of readmission on index admission, thereby reducing the overall readmission rates, healthcare utilization and inpatient mortality.

## Strengths and limitations

This study has several strengths and limitations. The study population, which is derived from one of the large, multi-ethnic hospital-based databases in the US, is believed to be a key strength of this study. Though our unique analysis, we examine numerous outcome-oriented facets for index admissions and 30-day readmissions of CDE. However, as with any study, this study is not exempt for limitations. The data available from the NRD is subject to biases associated with retrospective studies such as selection bias (all hospitalized patients) and the innate ability to discern patterns in seemingly random sets of data, as recognition of a pattern prior to analysis [23]. Hence, our study is meant to be a means to encourage future large, prospective, multicenter studies on the disease entity to further investigate our findings. Additionally, the NRD contains hospitalization data rather than data for individual patients. Thus, patients readmitted numerous times would be included more than once in our dataset. Moreover, the NRD lacks data on the time from hospital discharge after index hospitalization to readmission, severity of the disease, hospital course and the treatment aspects of the disease. Furthermore, like other administrative databases, there may be imitations related to reporting due to a lack of a financial incentive to document data. This limitation has been noted specifically with less costly and non-invasive studies, such as computed tomographic scans, ultrasound, and electrocardiography [23]. Our study does not investigate diagnostic procedures, and the limitations of the use of administrative databases regarding CDE are not clear; however, it is recognized that not all studies are affected by under-coding to the same degree [24]. The NRD also uses ICD-10 codes to report hospitalizations and thus may be prone to human coding errors. Finally, our study population had more females compared to males; however, previous retrospective studies have reported an equitable distribution of CDE with gender [25]. This may be due to an intrinsic limitation of this retrospective database. However, despite these limitations, we believe that the demographics studied, large sample size, and a unique analysis technique adds meaningful data on one of the major healthcare burdens in the US responsible for index admissions, readmissions and hospital acquired infections. Through this study, we aim to encourage further conversation and research on CDE readmissions.

## Conclusion

CDE places substantial burden on the US healthcare system in terms of costs and resource utilization. Patients admitted to the hospital for CDE, particularly those with multiple comorbidities, are at a higher risk of significant morbidity and mortality. In this study, we identified 94,668 index admissions and 18,296 readmissions at 30-days for CDE. The 30-day all-cause readmission rate for CDE was 25.7% with CDE being the most common principal diagnosis on readmission. Compared to index admissions, we noted higher odds of inpatient mortality (4.4 vs 1.4%, OR:3.32, 95% CI:2.87–3.84, *p* < 0.001), longer mean LOS (6.4 vs 5.6 days, MD: 0.9, 95% CI:0.7–1.0, *p* < 0.001), and higher mean THC ($56,015 vs $40,871, MD:15,144, 95% CI:13,260–17,027, *p* < 0.001) for 30-day readmissions of CDE. Independent predictors for 30-day all-cause readmissions of CDE included being discharge AMA, DM, and CKD. Additional large, prospective, multi-center studies are needed to further confirm these findings.

## Author contributions

Asim Kichloo, Zain El-Amir and Dushyant Singh Dahiya are credited with substantial contribution to the design of the work, acquisition, and interpretation of the data, drafting the manuscript, revision of important intellectual content, final approval of the version published, and agreement of accountability for all aspects of the work. Mohammad Al-Haddad, Jagmeet Singh, Gurdeep Singh and Carlos Corpez are credited with substantial contribution to interpretation of data, literature review of all sections discussed, final approval of the version published, and agreement of accountability for all aspects of the work. Hafeez Shaka is credited with analysis of the data, interpretation of the data, literature review of all sections discussed, final approval of the version published, and agreement of accountability for all aspects of the work.

## Data Availability

The NRD is a large, multi-ethnic, all-payer publicly available inpatient database containing information on more than 18 million hospital stays per year in the US. Its large sample size provides sufficient data for analysis of readmissions for both common and relatively uncommon disorders across all hospital types. The NRD is available at: https://www.hcup-us.ahrq.gov/nrdoverview.jsp.
